# Can We Foster a Culture of Peer Support and Promote Mental Health in Adolescence Using a Web-Based App? A Control Group Study

**DOI:** 10.2196/mental.5597

**Published:** 2016-09-23

**Authors:** Laura Bohleber, Aureliano Crameri, Brigitte Eich-Stierli, Rainer Telesko, Agnes von Wyl

**Affiliations:** ^1^ School of Applied Psychology Zurich University of Applied Sciences Zurich Switzerland; ^2^ Institute for Information Systems School of Business University of Applied Sciences Northwestern Switzerland Olten Switzerland

**Keywords:** mental health, health promotion, mobile applications, adolescence, peer group, mentors

## Abstract

**Background:**

Adolescence with its many transitions is a vulnerable period for the development of mental illnesses. Establishing effective mental health promotion programs for this age group is a challenge crucial to societal health. Programs must account for the specific developmental tasks that adolescents face. Considering peer influence and fostering adolescent autonomy strivings is essential. Participation in a program should be compelling to young people, and their affinity to new technologies offers unprecedented opportunities in this respect.

**Objective:**

The Companion App was developed as a Web-based app giving adolescents access to a peer mentoring system and interactive, health-relevant content to foster a positive peer culture among adolescents and thereby strengthen social support and reduce stress.

**Methods:**

In a control group study design, a group of employed (n=546) and unemployed (n=73) adolescents had access to the Companion App during a 10-month period. The intervention was evaluated using a combination of quantitative and qualitative approaches. Linear mixed effects models were used to analyze changes in chronic stress levels and perception of social support. Monthly feedback on the app and qualitative interviews at the end of the study allowed for an in-depth exploration of the adolescents’ perception of the intervention.

**Results:**

Adolescents in the intervention group did not use the Companion App consistently. The intervention had no significant effect on chronic stress levels or the perception of social support. Adolescents reported endorsing the concept of the app and the implementation of a peer mentoring system in particular. However, technical difficulties and insufficiently obvious benefits of using the app impeded more frequent usage.

**Conclusions:**

The Companion Project implemented a theory-driven and innovative approach to mental health promotion in adolescence, taking into account the specifics of this developmental phase. Particularities of the implementation context, technical aspects of the app, and insufficient incentives may have played considerable roles concerning the difficulties of the Companion Project to establish commitment. However, adopting peer mentoring as a strategy and using an app still seems to us a promising approach in mental health promotion in adolescents. Future projects should be careful to invest enough resources into the technical development of an app and consider a large use of incentives to establish commitment. When targeting risk groups, such as unemployed adolescents, it may be expedient to use more structured approaches including face-to-face support.

## Introduction

### Fostering Adolescent Health

There is now substantial evidence that thoughtfully designed health promotion programs for adolescents can be efficient and cost effective [1-3]. However, such programs must account for the specific developmental challenges adolescents face and carefully consider their expectations and needs [4]. We advocate that this implies accounting for the importance of peer influence and the adolescent drive for autonomy. Moreover, program participation needs to be compelling to young people.

### Including Peers and Fostering Empowerment

Adolescents must navigate many biological, cognitive, and psychosocial transitions. Most of all, they have to confront themselves with who they are and who they want to be [5,6]. Distancing themselves from parental ties, adolescents find new identifications and role models within their peer group [7,8]. Given the crucial importance of peer influence in adolescence [9-11], prevention programs should consider targeting peer groups as a whole. It is with their peers that adolescents negotiate health-related behaviors and attitudes. While research on peer groups has long focused on their role in reinforcing maladaptive behavior (eg, substance use), the peer group is also a place where self-enhancing and healthy behavior can be reinforced. In the 1980s, Vorrath and Brendtro [12] developed the concept of a positive peer culture by assuming that young people need to identify with positive values, such as caring for their peers and helping them thereby improving their self-worth, feeling significance, and enhancing responsibility. Similarly, the positive youth development perspective has gained importance in adolescent research, stressing the resources and potentials of young people [13,14]. There are a number of preventive intervention programs that built on these ideas with considerable success [15-18]. Most programs aimed at promoting emotional and social core competencies such as self-determination, confidence in oneself and the future, engagement for others, and prosocial bonding. Many encouraging findings of these studies expand upon insights gained during decades of research on the protective role of social support for mental (and physical) health [19-23].

Health promotion programs for young people need to provide for their desire to act and feel autonomously. Adolescents strive for a sense of self-governance, self-reliance, and individuation [8]. Adopting participatory approaches such as those advocated in youth empowerment approaches are promising in this respect [24,25]. Young people do not want to be addressed as passive recipients in health promotion programs; they want to be respected as active agents with sophisticated knowledge on health relevant issues and the socioeconomic environment in which they live [26]. Programs must account for the adolescents’ perspective on their health and how they construct their world [4,27]. Ideally, this should be the foundation of the development and implementation of a mental health promotion program.

Peer approaches account for both the essential influence of peers on the adolescent individual behavior and the adolescent drive for autonomy. They build upon existing expertise among adolescents and their specific view on health-related issues. There is a growing body of research on peer education in health promotion; examples exist in the field of HIV prevention [28] and substance use [29]. Peer mentoring has played a less prominent role in health promotion to this point, although we can refer to many insights from research on other forms of mentoring [30,31]. In fact, peer mentoring appears particularly attractive for health promotion efforts that embrace a holistic approach and do not target prevention of a specific pathology [32]. Karcher [33] demonstrated the usefulness of cross-age peer mentoring in a program with high school and elementary school students in a randomized trial. He showed the program enhanced feelings of connectedness to school and parents and that mentor attendance had a positive impact on several psychosocial outcomes in mentees. Results such as these are promising and may also be transferable to other contexts such as adolescents in transition to work life.

### Choosing an Attractive Medium to Reach Young People

The means of program delivery is an important aspect of health promotion efforts in adolescence. Research suggests that the use of new media provides powerful tools for health promotion programs. Adolescents in Switzerland and other high-income economies show a great affinity for smartphones and social media; 97% of Swiss 12- to 19-year-olds and 83.7% of American 13- to 17-year-olds own a smartphone [34,35]. Mobile’s share of Internet use is steadily growing [36], and apps are becoming more dominant: 86% of the time on American mobile devices is spent on apps [37]. New technologies supply novel forms of participatory communication that may render health promotion programs more attractive to young people. During the last decade, a growing number of studies have implemented new technologies in mental health promotion and prevention programs in adolescents [38]. Modalities such as cognitive behavioral therapy (CBT)–based Internet programs, Internet-based education programs, psychoeducational websites, online professional support and self-help groups and forums, counseling chats, Internet group therapy in chat rooms, and Internet-based games have been used to promote health and prevent mental disorders. Interventions are aimed at healthy adolescents, adolescents at risk, and adolescents with psychiatric symptoms, mostly in the context of mood disorders or disturbed social functioning. It remains difficult to judge the overall effectiveness of such interventions. Study designs are heterogeneous, and their quality varies to a great extent. There is growing evidence for the effectiveness of CBT-based programs for mood disorders [39]. Other approaches still need to strengthen their evidence base. Specifically, we know little about the effectiveness of peer approaches using new media. Two recent interventions integrated such an approach but could not show any effects of their intervention [40,41]. Few interventions have explored the advantages of implementing a project via an app [42], although there has recently been an effort to clarify the premises of using smartphone apps in mental health care and prevention [43].

The Companion Project took on this challenge and developed an app (the Companion App) aiming at fostering peer support and reducing stress in adolescents.

### The Context and Aims of the Companion Project

#### Being an Adolescent in Switzerland

After lower secondary education, two-thirds of Swiss adolescents start work life with an apprenticeship. On average, they are 15.5 years old when leaving lower secondary education [44]. This requires choosing a profession and beginning an adult-life work rhythm at a young age. In a recent study on stress levels in the Swiss working population, Grebner et al [45] found not only that chronic stress is a widespread issue but also that young workers (aged 15 to 24 years) experience higher stress levels than their older colleagues. Many Swiss apprentices report being markedly exposed to the experience of stress [46] and desire support in this respect [47]. Furthermore, adolescents who do not manage the transition from school to a profession are a particularly vulnerable group suffering from psychological distress [48,49]. Given the major mental health impairments that may result from unemployment at this age [50], these young people should be given special attention. So far, no specific stress reduction or mental health promotion strategies have been implemented for adolescents taking their first paths into work life in Switzerland nor for those failing to do so. The Companion Project fills in this gap.

#### Aims and Hypotheses

The Companion Project pursued a global approach to promote mental health in adolescents taking their first paths into work life as well as for those failing to do so. We proposed that the peer mentoring system, increased communication via the Companion App, and informational elements of the app would contribute to a positive peer culture among users. We supposed that enhanced social support through peers would globally promote the well-being of the adolescents and specifically impact experienced stress levels. Correspondingly, we hypothesized that the intervention via the Companion App would influence stress levels (reduce chronic stress) and perceived social support (augment perceived social support) in the adolescents of the intervention group compared to controls.

## Methods

### Overall Study Design

**Figure 1:**
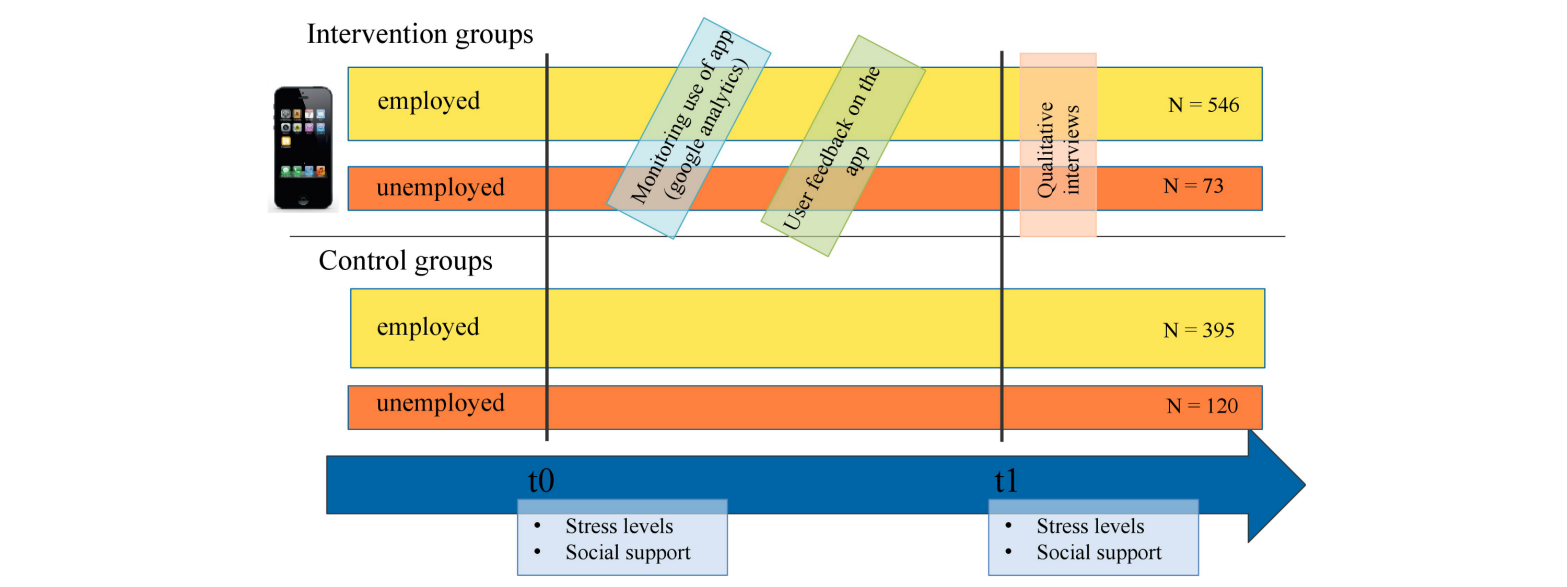
Control group study design.

The Companion App was developed based on the theoretical premises described above and as part of a mental health promotion effort initiated by Health Promotion Switzerland [51]. Health Promotion Switzerland aimed at developing a specific intervention targeting employed (in an apprenticeship) and unemployed youth. The conception and features of the Companion App were developed by the study team and discussed with the target group. Our information technology specialist team ran the technical implementation. The Companion App was then tested in a 10-month study using a control group design (Figure 1). The Companion App was introduced to the adolescents in a group presentation at the beginning of the study. Special attention was drawn to the peer mentoring system during that presentation. Throughout the study, the frequency of use of the Companion App was monitored: access to the app was tracked using Google Analytics. Importantly, the research team did not access the content users put on the app (eg, messages) because anonymity was guaranteed. Several incentives were employed to stimulate use of the app, such as monthly emails reminding the adolescents of the app and the peer mentoring systems and the distribution of flyers and posters. Users were invited to answer a monthly survey on the app concerning their use and suggestions for improvement. Doing so, they could participate in a lottery. Stress levels and perception of social support were evaluated before and after the intervention. Qualitative interviews at the end of the project explored the adolescent perceptions of the intervention and the Companion App.

### Recruitment

For the employed group, workers in their first and second year of apprenticeship at a large Swiss company were recruited for the intervention and control groups. In the intervention group, all apprentices across professions and sites of the company had access to the Companion App (n=546). The control group received no intervention and was recruited from different regional sections of the same company (n=395). For the unemployed adolescents, the intervention group was recruited from a publically financed transitional program for unemployed youth (n=73). The control group was, again, recruited from a different regional section of the same type of transitional program (n=120). We based our recruitment on an estimation of the necessary sample size to detect a small-to-medium effect size at a 95% confidence level according to Cohen [52]. Approximating the statistical models we used in order to explore changes in chronic stress and the perception of social support, we assumed a regression model with 6 predictors. Given such a model, a sample size of 200 is sufficient to detect an effect of *R*^2^=.07 with a power of .83.

### Instruments

Stress was measured using the Trier Inventory of Chronic Stress (TICS) [53], a 57-item scale tapping different stress dimensions including work overload, social overload, pressure to perform, work discontent, excessive demands from work, lack of social recognition, social tensions, social isolation, and chronic worrying. All items are rated on a 5-point Likert scale (0 = never, 1 = rarely, 2 = sometimes, 3 = often, 4 = very often). Answers refer to how often participants had an experience during the last 3 months. The Trier Inventory of Chronic Stress screening scale (TICS-SCSS) uses 12 of the most salient items of the other dimensions and provides a global index for chronic stress. In our sample, all scales of the TICS showed good to excellent internal consistency (Cronbach alpha range .80-.92).

Satisfaction with social support and reciprocity in social support were captured using two scales of the social support questionnaire (Fragebogen zur sozialen Unterstützung, F-Soz-U) [54]. Whereas satisfaction with social support using four items showed good internal consistency (Cronbach alpha=.83), the internal consistency of reciprocity in social support using four items was questionable (Cronbach alpha=.63). All items are rated on a 5-point Likert scale (0 = strongly disagree to 4 = strongly agree).

### Procedures

We evaluated stress and perception of social support before and after the intervention. In the employed group, questionnaires were administered in paper-and-pencil form at the beginning of the year of apprenticeship (t0) and at the end (t1). For apprentices in their second year of apprenticeship, the t1 evaluation was done using an online survey. In the unemployed group, evaluations took place when the young people first joined the transitional program (t0) and when they left (t1). They filled out paper-and-pencil questionnaires. In general, adolescents leave the transitional program once they find an apprenticeship, an internship, or another form of employment.

At the end of the study, we also conducted semistructured interviews with 6 employed adolescents and 8 unemployed adolescents for an in-depth exploration of their perceptions of the Companion Project.

### The Companion App

The concept of the Companion App was discussed with adolescents taking their first paths into their working life. Five school classes with 10 to 15 apprentices aged 15 to 17 years participated in focus groups and discussed their expectations and needs concerning a mental health promotion intervention.

Core features of the Companion App:

- Peer mentoring system: every Companion App user had a mentor who was in a similar professional situation. The peer mentoring system was presented to all of the app users in the beginning of the intervention. Emails explaining the mentoring system and reminding the Companion App users of it were sent once a month during the intervention. In the employed group, mentees and mentors were associated by the research team based on their apprenticeship and location. In the unemployed group, the mentoring system was looser. App users could ask other users on the app to become their mentor.
- Individual profile for each user with the possibility to upload pictures and mention specific interests
- Messaging and discussion groups
- Links to interactive and informative websites on mental health-related issues (eg, psychological tests, sexuality, drug use)
- Links to websites concerning leisure activities (eg, tips for going out on the weekend)
- An anonymous professional counseling service (run by a psychologist or social worker) including a blog run by the professional with posts on diverse topics

**Figure 2:**
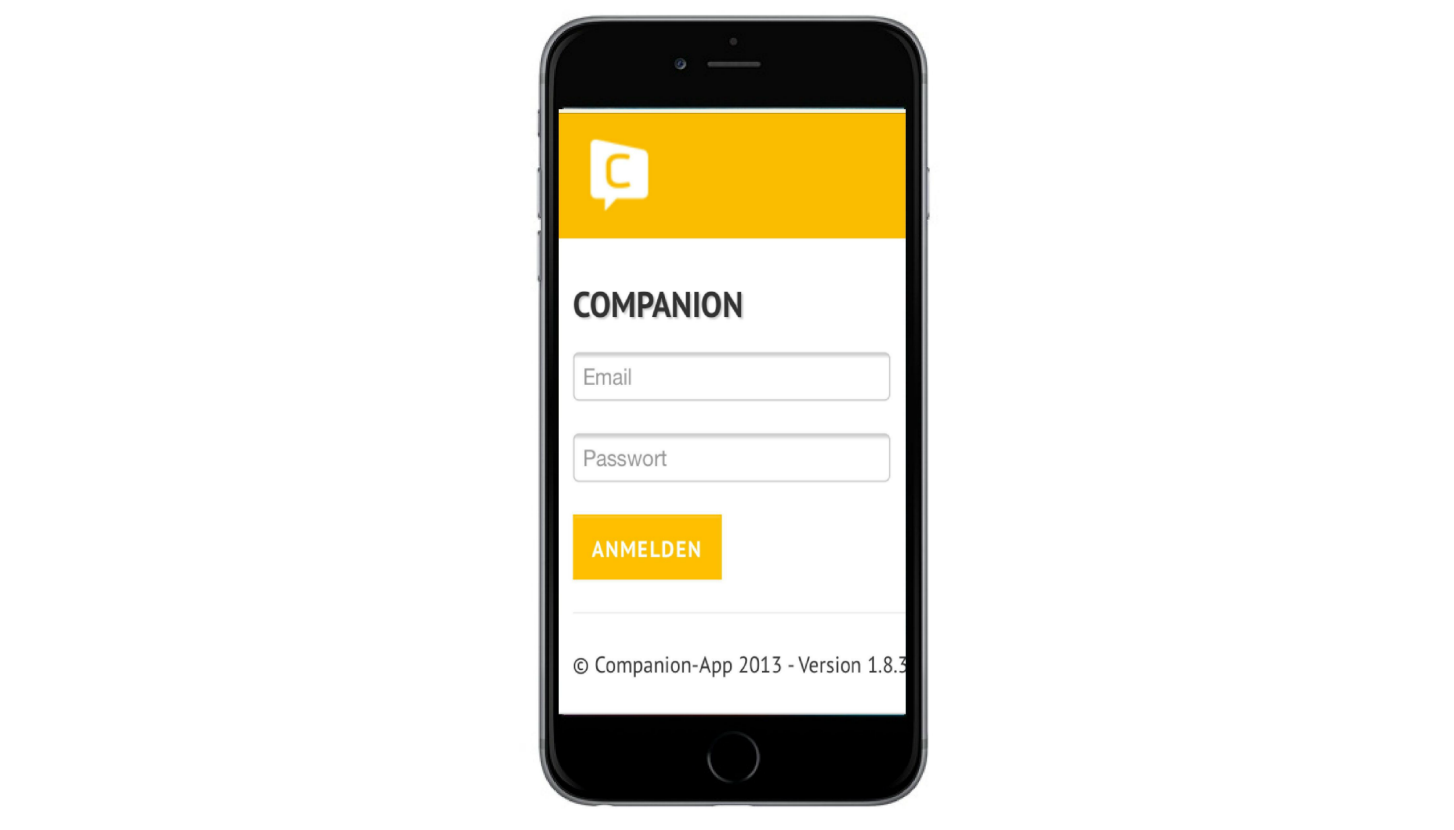
Design of the companion app.

The Companion App (see Figure 2) was programmed as a Web-based app so users could access it from either a smartphone or a computer.

During the Companion Project, all users had the option to give monthly feedback on their use of the Companion App and to communicate suggestions for improvements or new features. Frequently mentioned suggestions consistent with the app’s concept were then implemented in the app. For instance, some users asked for the option to have a status on their profile, where they could describe current activities. A corresponding feature was added during the study.

### Analyses

Descriptive statistics were used to describe the sample, and all statistical analyses were conducted using SPSS 22 (IBM Corp). Monthly surveys on the frequency of use, perception of the app, and suggestions for improvement were analyzed regrouping answers into thematic categories. Access to the app was recorded using Google Analytics. Qualitative interviews were conducted using a semistructured interview outline. Interviews were coded and answers categorically regrouped using the qualitative data analysis software MAXQDA (Verbi GmbH).

Multiple linear regression was used to analyze stress levels and perception of social support. Status of employment (employed vs unemployed), gender, nationality (Swiss vs non-Swiss), and age were used as predictors while stress and the perception of social support were dependent variables in separate regression models.

Linear mixed models were then employed to investigate changes in stress levels and the perception of social support during the Companion Project. These models allow analyses of data with repeated measures estimating changes at the individual as well as at the group level. To estimate differences between control and intervention groups and changes over time (group-time interaction), sets of predictors were used in different models and compared with likelihood-ratio tests. The sequence of predictor sets is described in the results section.

## Results

### Participants

In the employed group, 477 apprentices in their first year participated in the t0 (return rate of 477/492, 97.0%) and 443 in t1 evaluation (return rate of 443/492, 90.0%). Evaluations were anonymized. Apprentices filled out a personalized but anonymous code on their questionnaires (first letter of the first name of mother and father and the sum of their date of birth). Due to many errors in these codes, only 65.9% (292/443) of the questionnaires could be matched (matching code from t0 to the one of t1). A total of 464 apprentices in their second year participated in the t0 (return rate of 464/472, 98.3%) and 226 in the t1 online evaluation (return rate of 226/472, 47.8%). More than two-thirds (156/226) of the t1 questionnaires could be matched to the corresponding t0 questionnaires.

In the unemployed group, 193 adolescents participated in the t0 and 43 in the t1 evaluation. We estimated that return rates were 43% for t0 and 10% for t1. Because of the small number of t1 evaluations, the data of this group was not analyzed longitudinally.

In the employed group, age was on average 16.9 (standard deviation [SD] 1.73) years in the first year of apprenticeship and 17.6 (SD 1.46) years in the second. A total of 50.2% and 56.7% of the participants were female in the first and second years, respectively.

In the unemployed group, participant average age was 18.4 (SD 1.96) years, and 40.4% of the participants were female.

### Use of the Companion App

Google Analytics revealed that in the first two weeks of the study, the app had 61 daily visits on average with a peak of 189 visits the day after we sent out the log-in details to the adolescents. Six months later, halfway through the project, daily visits over two weeks averaged 8. A significant augmentation in use was not achieved until the end of the study.

### Companion App User Feedback

In the employed intervention group, adolescents were invited to evaluate their use and satisfaction with the app 8 times. On average, 34.4 adolescents participated in these evaluations. Evaluation 1 had the most participants, with a total of 60.

In evaluation 1, the most frequently identified reasons for using the app were curiosity and interest in a new app (13 answers, regrouped into this thematic category) and getting to know other apprentices (14 answers). Participants also mentioned that they used the app because of the peer mentoring system (6 answers) and for the psychological tests that they could take on the app (4 answers).

The most frequently mentioned reasons for not using the app more often were that participants could not see the benefits of using the app (14 answers) and lack of time (12 answers). Moreover, participants mentioned that they did not use the app more frequently because there was not enough activity by other users (5 answers) and there had been technical difficulties (3 answers).

Participants suggested making the design of the app more attractive (eg, improve the layout, create a more intuitive structure; 8 answers) and rectifying technical issues (7 answers). Some participants suggested rendering the app more interesting by creating supplementary contents such as games or by connecting the Companion App to other social media or existing Web platforms of the company (5 answers).

### Qualitative Interviews

Qualitative interviews were conducted with both employed (n=6) and unemployed adolescents (n=8) in the intervention group for an in-depth analysis of their perceptions of the Companion App project (Table 1).

The majority of the interviewees in both groups regarded the concept of the app as well conceived. For instance, participant 4 said, “I like the basic idea, the idea of helping one another and getting feedback from a friend instead of an authority.” Equally, almost all of the interviewees judged that a peer mentoring system would be helpful to them. Whereas the majority of employed adolescents reported that they would ask a mentor questions about organizational aspects of their apprenticeship, the unemployed adolescents said that they would turn to a mentor for advice on how to best find an apprenticeship and how to perform best during a working trial. For example, participant 5 said, “I would ask [my mentor]: How did you do it [find an apprenticeship], what was your approach? How did you pull through, where did you find the energy for that?”

Concerning the design of the app, the most common feedback from the employed adolescents was that they thought it was well structured and visually attractive. The content was judged informative and interesting. Most interviewees said that the Companion App would offer them something specific that existing commercial apps could not provide. However, all interviewees judged that the app was not competitive compared to existing apps on the market.

Both employed and unemployed adolescents named technical problems as the main reason for not using the app more frequently. Some of them had forgotten their log-in details and did not put any effort into getting a new log-in, as illustrated by participant 10’s statement: “The only reason I didn’t use it was that I forgot my password.” Additionally, interviewees reported lacking interest and not knowing the purpose of the app as further reasons for not using it. In the employed group, three adolescents also said that they communicated on other social media platforms. Moreover, two interviewees named a missing user base as a reason for not using the app more frequently.

The two groups differed substantially in their suggestions on how to improve the app. Whereas the majority of employed adolescents recommended easier and more intuitive handling of the app, the majority of unemployed adolescents suggested that the app should be promoted more intensively. In line with that, unemployed interviewees also mentioned that it would have been a great help to them to be reminded more frequently to use the app.

In sum, all of the interviewed adolescents who used the Companion App rated the content and design as attractive and well conceived. All interviewees appreciated the idea of having a peer mentor and being able to ask him or her for advice on aspects of their professional situation. They mentioned that the handling of the app should be improved and technical problems resolved. Unemployed interviewees reported that it would have been a help to be reminded more frequently and that the app should have been promoted more intensively.

**Table 1:** Selected quotes from interviews regrouped into thematic categories.

Category	Subcategory	Example
**Perception of the app**		
	Design	I think the app is professionally designed. [Participant 11]
	Content	Lots of information and diverse topics. [Participant 1]
	Handling	The handling is inconvenient [...]. [Participant 12]
**Obstacles to frequency of use**		
	Technical issues	My password wasn’t valid, and I didn’t try again. [Participant 6]
	No interest	I didn’t want to download the app. [Participant 7]
	Purpose uncertain	The app was briefly explained, but I didn’t entirely get it. [Participant 3]
	Missing user base	[I did not use the app more often]...because my friends didn’t use it. [Participant 13]
	Other networks used	Since there are Facebook and other platforms, those are used more frequently. [Participant 10]
**Areas of improvement**		
	Easier handling	Faster access. [Participant 12]
	Extended promotion	Promotion should be extended, more precise and more attractive information. [Participant 2]

### Stress and Social Support

Stress levels and the perception of social support were explored before and after the intervention in a control group design. Here, we first report our findings at baseline and then those on changes in stress levels and the perception of social support throughout the study.

At baseline, employed adolescents of the intervention group (539/964) showed a mean score of 1.25 (SD 0.64) on the TICS-SCSS assessing chronic stress. This means that on average these adolescents had rarely experienced chronic stress during the last 3 months. The mean of perceived social support (measured with the F-Soz-U) was 2.74 (SD 0.85) implying that employed adolescents were often satisfied with the social support they received.

Unemployed adolescents of the intervention group (73/193) showed a mean score of 1.37 (SD 0.66) on the TICS-SCSS at baseline, implying that on average they also had rarely experienced chronic stress during the last three months. On the F-Soz-U, they showed a mean of 2.63 (SD 0.92) indicating that they were often satisfied with the social support they experienced.

We used multiple linear regression to explore differences in chronic stress and the perception of social support considering the status of employment, gender, nationality (Swiss vs non-Swiss) and age. Regarding chronic stress, employment and age did not significantly predict this dimension, while gender and nationality did (see Table 2). However, the variance explained by the model was small, with *R*^2^=.04, *F*_4,549_=5.73, *P*<.001. Visual inspection of residual plots revealed no obvious deviations from homoscedasticity or normality.

An equivalent model was applied regarding perceived social support. Age and gender but not employment or nationality predicted perceived social support (see Table 3). The variance explained by the model was, again, small with *R*^2^=.025, *F*_4,528_=3.36, *P*=.01. Also, visual inspection of residual plots revealed slight deviations from homoscedasticity and normality.

**Table 2:** Multiple linear regression by chronic stress (employment reference category—employed, gender reference category—female, Swiss reference category—Swiss).

Predictor	B	SE B	β	T	*P* value
Constant	1.311	.040	—	33.063	.000
Employment	.081	.092	.038	.876	.381
Gender	−.223	.054	−.177	−4.158	.000
Swiss	.138	.062	.094	2.234	.026
Age	.016	.015	.046	1.070	.285

**Table 3:** Multiple linear regression by perceived social support (employment reference category—employed, gender reference category—female, Swiss reference category—Swiss).

Predictor	B	SE B	β	T	*P* value
Constant	2.653	.055	—	48.325	.000
Employment	.089	.128	.031	.696	.487
Gender	.191	.075	.112	2.556	.011
Swiss	−.076	.087	−.037	−.866	.387
Age	−.061	.021	−.131	−2.943	.003

For the group of employed adolescents, linear mixed effects analyses were performed to explore changes in stress levels and perception of social support before and after the intervention.

We used the TICS-SCSS to track changes in chronic stress levels. In a first step, we entered general predictors (time, gender, year of apprenticeship, and age) as fixed effects in a linear mixed model (see model A in Multimedia Appendix 1). Age was mean centered. We took intercepts for participants as random effects. In subsequent steps, we added group (intervention vs control, see model B in Multimedia Appendix 1) and the interaction between group and time (see model C in Multimedia Appendix 1) as fixed effects. Likelihood ratio tests were carried out to compare the deviances (*D*) of these models. Comparing model B (*D*=1575.98) to A (*D*=1572.46) indicated that adding group as predictor did not significantly improve model fit (*P*=.06). Comparing model C (*D*=1579.24) to B (*D*=1575.98) revealed that adding the group and time interaction did not significantly improve model fit (*P*=.07). Thus, no significant effect was found for group and the group and time interaction.

We inspected changes in perception of social support with equivalent models to those used for the analyses of stress levels (see Multimedia Appendix 2). The comparison of model E (*D*=2006.83) and D (*D*=2004.57) revealed no significant improvement in model fit (*P*=.13) when adding group as a predictor. Comparing model F (*D*=2007.32) and E indicated that adding the group and time interaction did not contribute to any better model fit (*P*=.49). Thus, there was again no significant effect of group and the group and time interaction on perception of social support.

For all models, visual inspection of residual plots revealed no obvious deviations from homoscedasticity or normality.

In summary, the intervention via the Companion App did not have any measurable effects on chronic stress levels or the perception of social support among adolescents in the intervention group.

## Discussion

### Principal Findings

Our Companion Project implemented a mental health promotion program with adolescents in Switzerland using an app. At the core of the project was a peer mentoring system. For 10 months a group of employed (n=546) and unemployed (n=73) adolescents had access to the Companion App. Effects of the intervention on stress levels and perception of social support were assessed in a control group study design. Monitoring of the use of the app showed that the adolescents did not use the Companion App frequently. We found no effects of the intervention on stress levels and perception of social support.

Here, we discuss our findings of the qualitative evaluations as well as the quantitative findings on stress levels and perception of social support. Most importantly, we consider reasons why the Companion App struggled to achieve solid utilization. Finally, we review what we can learn from the Companion Project and formulate recommendations for future intervention projects using apps in adolescent health promotion.

### Findings From Qualitative Interviews With the Adolescents: Interest But Insufficient Incentives and Unsatisfactory Technical Development

The Companion App failed to achieve solid participation. While in the first 2 weeks of the study daily visits averaged around 60, these numbers decreased markedly in later weeks. Qualitative interviews and feedback on the app by users gave us some hints on reasons for this.

Adolescents reported being interested in discovering a new app and that the content and design of the Companion App was attractive and well conceived. Moreover, the peer mentoring system was judged helpful. This suggests that the concept of the Companion App was essentially well received.

However, some adolescents reported that the benefits and purpose of the Companion App had not been evident to them. Some unemployed adolescents said they would have appreciated a more intense promotion of the app. It may be that the communication around the app and the incentives for use provided during the study were insufficient. We know that developing and maintaining engagement with an app is challenging [55]. We presented the app to the adolescents at the beginning of the study and sent emails throughout the study to the users informing about the app and the mentoring system. We also distributed flyers and posters in common spaces used by the adolescents of the intervention group. However, it seems that these efforts were insufficient.

According to the feedback of the adolescents, technical problems also hindered more frequent use of the app. This surely is a key factor. The Companion App was developed within the frame of a research project. Therefore, available resources for the technical implementation were limited. Time constraints limited some steps of the development process required for launching an app [56]. The app was technically improved during the study, but it may be that adolescents stopped attempting to use the app after the first experience of technical difficulties. We know that only a few users will try an app again once they have experienced technical failure [57,58].

### Findings on Social Support and Chronic Stress: No Wish for More and No Need for Less?

We will now discuss findings of the evaluation of chronic stress and the perception of social support. Because the Companion App struggled to achieve solid utilization and our analyses did not reveal significant changes in stress levels or perception of social support before and after intervention, we focus on discussion of the baseline results.

At baseline, we found that the adolescents in our intervention group were often satisfied with the social support they received and that they had rarely experienced chronic stress during the previous three months. Does this imply insufficient interest in an intervention of the type implemented by the companion project?

It may be that the overall satisfaction with social support in our sample led to a weaker interest in the peer mentoring system. This would, however, contradict some of our findings in the qualitative evaluations in which adolescents reported endorsing the implementation of a peer mentoring system. Also, from a health promotion perspective, one can argue that social support still can be enhanced or sustained respectively.

Concerning the experience of stress in our sample, can it be that stress was not a relevant matter for these adolescents? This would diverge from what we know from other studies. Jeannin and colleagues [47] asked apprentices and students in Switzerland in a representative sample (ages 16-20 years, n=7428) in which health domains they would like to receive support. More than one-quarter of the male and almost half of the female participants reported desiring support concerning the experience of stress. Padlina et al [46] found in their study that 64.3% of Swiss apprentices (n=211) reported they felt generally overburdened and stressed. We also compared the stress levels of the employed group of our sample to the German TICS norm sample for a similar age group (16-30 years, n=146) [53]. In the employed group, a mean of 1.25 (SD 0.64) and sum score of 14.97 (SD 07.65) on the TICS-SCSS corresponded to a *t* value of 52. This implies that mean scores of chronic stress of the employed adolescents in our sample are comparable to those of other adolescents and young adults. We do not have data collected using the same instrument (TICS-SCSS), so we cannot do a direct comparison to other Swiss apprentices.

We can, however, reflect several aspects of why stress levels in our sample may have been low. We should give consideration to the choice of our stress measurement instrument and the environment of our sample. The TICS-SCSS scale specifically measures chronic stress, thereby capturing severe and enduring experiences of stress. It is possible that by focusing on this screening scale, we left aside experiences of more mild forms of stress that may still be of importance to the adolescents. Additionally, lower stress levels in our sample may be linked to certain aspects of the organization (eg, preexisting structures such as an informal buddy system). We discuss these specifics in more detail in the subsequent part of the discussion.

Still, it is important to point out that despite the possibility that stress levels were lower in our sample compared to other apprentices, the Companion Project is a health promotion project. Therefore, in a preventive perspective, participation should also appeal to those adolescents who do not necessarily suffer from elevated stress levels.

Taking a closer look at the stress levels in the unemployed group of our study, we were surprised that they did not experience significantly higher stress levels than the employed adolescents. Also, their mean of 1.38 (SD 0.66) and a sum of 16.51 (SD 7.81) on the TICS-SCSS corresponded to a *t* value of 54 compared to the aforementioned German TICS norm sample. This was surprising to us, as we know that mental health of unemployed youth is often eroded and that the experience of stress is common [59]. For example, in a representative sample of 100 unemployed 16- to 24-year-olds, Reissner and colleagues [48] found that 43% presented a mental illness often associated with high stress levels. In Switzerland, Sabatella and von Wyl [49] found that 74% percent of the 151 unemployed adolescents they interviewed in state-funded transitional programs showed signs of psychological distress. The way we recruited the unemployed adolescents may explain why we found relatively low stress levels in our sample and why these did not differ from the employed group. The participants of our study were recruited from a state-funded transitional program, and our evaluation took place when they entered the program. Thus, the situation of being unemployed was still new to most of them. Also, participating in the transitional program likely provided a perspective of having a structure in daily life and of receiving support to find an apprenticeship or a job. Finally, the same considerations regarding our measurement scale of chronic stress not capturing milder forms of stress mentioned before apply to this group. Regardless of these findings, we should keep in mind that early prevention in this group of youth is of major importance, because unemployment seems to affect mental health and vice versa [60,61].

### Difficulties in Establishing Commitment to the Companion Project

We have already presented a few reasons provided by the adolescents in the qualitative evaluation for the infrequent use of the Companion App. We discuss additional considerations here.

First, specifics of the implementation context may have played a meaningful role for the nonadoption of the app by the users. Employed participants were recruited from a large Swiss company and unemployed participants from a state-funded transitional program. In the large Swiss company, the project was not directly adopted as a part of the company’s own health promotion strategy. This likely had an influence on the adherence to the project. For example, the information flow concerning the Companion App within the company environment may not have been sufficient. Also, the company had sophisticated preexisting structures to promote mental health amongst their employees. Although these were not specifically conceived to target their apprentices, it was not obvious how the Companion Project would integrate with these structures. Moreover, for apprentices, a sort of a natural buddy system already existed within the company. Apprenticeship positions were manned each year. The apprentice having occupied the position the year before was in charge of introducing the new apprentice. These preexisting structures may have contributed to the Companion App not signifying much additional benefit to the apprentices.

In the state-funded transitional program for unemployed youth, organizational structures were very flexible. Adolescents started and left the program throughout the duration of the study. The lynchpin to reach the adolescents in these programs was the social worker population. Each adolescent had one social worker responsible for him and accompanying him throughout the program. These social workers introduced the adolescents to the Companion App upon entering the program and also followed up on the adolescent’s participation. Time resources of the social workers were, however, limited. Unemployed adolescents mentioned in the qualitative interviews that they would have needed more reminders and promotion to use the app. We assume that a more intense follow-up and a sort of personal assistance in using the app would have been required. For example, some adolescents said that they had forgotten their log-in details and did not put any effort into getting a new log-in. As the social workers were not able to provide assistance of this type, implementing another form of personal follow-up may have been necessary. Obviously, such additional assistance would have augmented the costs of the Companion Project significantly. Generally, we presume that promoting health with unemployed adolescents needs to be more structured and that an intervention, including some face-to-face follow-up, may enhance their program adherence.

Second, we consider if the choice of implementing the Companion Project using an app was appropriate. Given the widespread use of mobile phones, the Internet, and specifically mobile phone apps [34-36], the choice to use an app seemed opportune. Also, this choice made the intervention less costly and likely increased our reach to more adolescents compared to face-to-face intervention. This is the great advantage of mental health interventions using new technologies [39,62]. However, initiating commitment and maintaining engagement are preeminent challenges for online interventions, and dropout rates have been mentioned as a central issue in previous studies [38]. Especially with regard to the peer mentoring system, we must consider if implementation of a face-to-face mentoring system would have fostered participation in the Companion Project. In its implementation for this study, personal contact remained mediated by a technical device. Chatting with a mentor on a device is much different than meeting a mentor in person and likely creates a different level of commitment to the mentoring relationship.

Third, we examine our use of incentives. Incentives probably play a major role in prevention programs for adolescents [63]. Throughout the program we created different kinds of incentives to use the app (advertisement via posters and flyers, emails informing about the app, lotteries in which adolescents could participate when taking part in evaluation of the app), but apparently these were insufficient. Further, adolescents received little to no personalized monitoring concerning their use of the Companion App. They were introduced to the Companion App at the beginning of the program and received reminder emails throughout its course. However, there was no personal follow-up on their use of the app. Such follow-up may have enhanced their engagement with the app.

### Insights for Future Mental Health Promotion Programs in Adolescence Using an App

In summary, we can draw several insights from the Companion Project that may be useful to future mental health promotion projects with adolescents using an app.

First, we think that a theory-driven approach acknowledging the specific developmental challenges that adolescents face is recommended. Given the importance of peer influence at this age and the adolescent drive for autonomy, peer mentoring seems to offer a great opportunity to do so. The qualitative interviews we conducted with the adolescents revealed that adolescents supported this concept. However, future projects should carefully consider if a peer mentoring system can be implemented with an app only and if face-to-face contact between mentors and mentees enhances adherence to the mentorship.

Second, we would like to emphasize the advantages of adopting a holistic approach to health promotion (versus focusing on the prevention of a specific pathology). There is evidence concerning the protective effects of social support regarding many domains of mental (and physical) health [19-23]. This evidence also takes into account that adolescents tend to engage in combinations of health-risking behaviors [64]. Peer mentoring offers a natural opportunity of implementing an holistic approach.

Third, using an app in a health promotion effort is promising. Given the frequent use of apps by wide parts of the population, and by adolescents in particular, apps have the potential to reach many people. However, we experienced first-hand the difficulties of establishing user commitment. We found that adolescent app users tend to be unforgiving if there are technical difficulties. Moreover, we realized that any app launched today enters a difficult competitive battle with many commercially developed apps. Therefore, establishing usability, feasibility, and accessibility and ensuring enough time for the technical refinement of the app is essential. Recently, efforts have been put into clarifying the requirements for apps in the mental health field [43]. We also propose that adopting a participatory approach by involving adolescents in the development of such an app is encouraging. This can enhance the adoption of the app in the respective target group.

Fourth, using well-chosen and sufficient incentives to strengthen commitment to the program seems essential. The form and use of incentives surely depends on the specific project. In the case of the Companion Project, more intensive promotion of the app and a personalized follow-up of the use of the app may have been necessary.

Finally, we think that certain groups of adolescents, especially a high-risk group as our intervention group of unemployed adolescents, likely benefit from more structured interventions. In the case of our project, providing these groups with additional face-to-face support to aid in discovering the Companion App and peer mentoring system may have been advantageous.

### Conclusion

The companion project implemented a theory-driven and innovative approach to mental health promotion in adolescence, taking into account the specifics of the adolescent developmental phase. The Companion App struggled to achieve solid utilization, and the intervention did not have any effects on chronic stress and the perception of social support. Nevertheless, the project generated insights on opportunities and pitfalls when using an app in a mental health promotion effort in adolescence and can inform future projects in the field.

## Appendix

Multimedia Appendix 1 Mixed models for the prediction of change in chronic stress levels from pre to post evaluation: Model A, B, and C.

Multimedia Appendix 2 Mixed models for the prediction of change in perception of social support from pre- to postevaluation: Model D, E, and F.

## Abbreviations

CBT: cognitive behavioral therapy
F-Soz-U: Fragebogen zur Sozialen Unterstützung
TICS: Trier Inventory of Chronic Stress
TICS-SCSS: Trier Inventory of Chronic Stress screening scale

## References

[1] Catalano RF, Fagan AA, Gavin LE, Greenberg MT, Irwin CE, Ross DA, Shek DT (2012). Worldwide application of prevention science in adolescent health. Lancet.

[2] D'Arcy C, Meng X (2014). Prevention of common mental disorders: conceptual framework and effective interventions. Curr Opin Psychiatry.

[3] Lee S, Aos S, Penucci A (2015). What works and what does not? Benefit-cost findings from WSIPP.

[4] Mackinnon D (2007). Health promotion and health education. Coleman J, Hendry LB, Kloep M. Adolescence and Health.

[5] Blos P (1979). The Adolescent Passage.

[6] Erikson E (1959). Identity and the Life Cycle: Selected Papers.

[7] Brown BB, Eicher SA, Petrie S (1986). The importance of peer group (“crowd”) affiliation in adolescence. J Adolesc.

[8] Zimmer-Gembeck M, Collins W, Adams GR, Berzonsky MD (2006). Autonomy development during adolescence. Blackwell Handbook of Adolescence.

[9] Brown B, Bakken J, Ameringer S, Mahon D, Prinstein M, Dodge K (2008). A comprehensive conceptualization of the peer influence process in adolescence. Understanding Peer Influence in Children and Adolescents.

[10] Brown B, Larson J, Lerner R, Steinberg L (2009). Peer relationships in adolescence. Handbook of Adolescent Psychology, 3rd edition.

[11] Lila M, von Aken M, Musitu G, Buelga S, Jackson S, Goossens L (2006). Peer relations in adolescents. Handbook of Adolescent Development.

[12] Vorrath H, Brendtro L (1985). Positive Peer Culture.

[13] Lerner RM (2005). Positive youth development a view of the issues. J Early Adolescence.

[14] Silbereisen R, Lerner R (2007). Approaches to Positive Youth Development.

[15] Durlak JA, Taylor RD, Kawashima K, Pachan MK, DuPre EP, Celio CI, Berger SR, Dymnicki AB, Weissberg RP (2007). Effects of positive youth development programs on school, family, and community systems. Am J Community Psychol.

[16] Gavin LE, Catalano RF, David-Ferdon C, Gloppen KM, Markham CM (2010). A review of positive youth development programs that promote adolescent sexual and reproductive health. J Adolesc Health.

[17] Guerra NG, Bradshaw CP (2008). Linking the prevention of problem behaviors and positive youth development: core competencies for positive youth development and risk prevention. New Dir Child Adolesc Dev.

[18] Lapalme J, Bisset S, Potvin L (2014). Role of context in evaluating neighbourhood interventions promoting positive youth development: a narrative systematic review. Int J Public Health.

[19] Auerbach RP, Bigda-Peyton JS, Eberhart NK, Webb CA, Ho MR (2011). Conceptualizing the prospective relationship between social support, stress, and depressive symptoms among adolescents. J Abnorm Child Psychol.

[20] Bal S, Crombez G, Van Oost P, Debourdeaudhuij I (2003). The role of social support in well-being and coping with self-reported stressful events in adolescents. Child Abuse Negl.

[21] Cohen S, Wills TA (1985). Stress, social support, and the buffering hypothesis. Psychol Bull.

[22] Taylor SE, Stanton AL (2007). Coping resources, coping processes, and mental health. Annu Rev Clin Psychol.

[23] Umberson D, Montez JK (2010). Social relationships and health: a flashpoint for health policy. J Health Soc Behav.

[24] Tengland P (2007). Empowerment: a goal or a means for health promotion?. Med Health Care Philos.

[25] Wong NT, Zimmerman MA, Parker EA (2010). A typology of youth participation and empowerment for child and adolescent health promotion. Am J Community Psychol.

[26] Shepherd J, Garcia J, Oliver S, Harden A, Rees R, Brunton G, Oakley A (2002). Barriers to, and facilitators of the health of young people: a systematic review of evidence on young people's views and on interventions in mental health, physical activity and healthy eating. Volume 2: Complete report.

[27] Shucksmith J, Spratt J (2002). HEBS Young People Health Initiative.

[28] Medley A, Kennedy C, O'Reilly K, Sweat M (2009). Effectiveness of peer education interventions for HIV prevention in developing countries: a systematic review and meta-analysis. AIDS Educ Prev.

[29] Georgie JM, Sean H, Deborah MC, Matthew H, Rona C (2016). Peer-led interventions to prevent tobacco, alcohol and/or drug use among young people aged 11-21 years: a systematic review and meta-analysis. Addiction.

[30] DuBois DL, Silverthorn N (2005). Natural mentoring relationships and adolescent health: evidence from a national study. Am J Public Health.

[31] Crosnoe R, McNeely C (2008). Peer relations, adolescent behavior, and public health research and practice. Fam Community Health.

[32] Steinebach C, Steinebach U, Kösler E (2006). Neue Wege der Jugendberatungvaluation von Positive Peer Culture (PPC). Forschen und Weiterbilden für eine Soziale Zukunft.

[33] Karcher MJ (2004). The effects of developmental mentoring and high school mentors' attendance on their younger mentees' self-esteem, social skills, and connectedness. Psychol Schs.

[34] Willemse I, Waller G, Genner S, Suter L, Oppliger S, Huber A, Suess D (2014). JAMES - Jugend, Aktivitaten, Medien - Erhebung Schweiz.

[35] Lenhart A (2015). A majority of American teens report access to a computer, game console, smartphone and a tablet.

[36] Bosomworth D Smart Insights.

[37] Khalaf S (2014). Android personalization apps: The battle for app, content and service discovery.

[38] Clarke AM, Kuosmanen T, Barry MM (2015). A systematic review of online youth mental health promotion and prevention interventions. J Youth Adolesc.

[39] O'Dea B, Calear AL, Perry Y (2015). Is e-health the answer to gaps in adolescent mental health service provision?. Curr Opin Psychiatry.

[40] Freeman E, Barker C, Pistrang N (2008). Outcome of an online mutual support group for college students with psychological problems. Cyberpsychol Behav.

[41] Horgan A, McCarthy G, Sweeney J (2013). An evaluation of an online peer support forum for university students with depressive symptoms. Arch Psychiatr Nurs.

[42] Dennison L, Morrison L, Conway G, Yardley L (2013). Opportunities and challenges for smartphone applications in supporting health behavior change: qualitative study. J Med Internet Res.

[43] Bakker D, Kazantzis N, Rickwood D, Rickard N (2016). Mental health smartphone apps: review and evidence-based recommendations for future developments. JMIR Ment Health.

[44] (2015). Swiss Federal Statistical Office.

[45] Grebner S, Berlowitz I, Alvarado V, Cassina M (2010). Stress in Swiss employees. Associations between working conditions, person characteristics, well-being, and health.

[46] Padlina O, Ceesay K, Gehring T (2002). Prevention of smoking and stress in adolescents.

[47] Jeannin A, Narring F, Tschumper A, Bonivento LI, Addor V, Bütikofer A, Suris J, Diserens C, Alsaker F (2005). Self-reported health needs and use of primary health care services by adolescents enrolled in post-mandatory schools or vocational training programmes in Switzerland. Swiss Med Wkly.

[48] Reissner V, Mühe B, Wellenbrock S, Kuhnigk O, Kis B, Dietrich H, Hebebrand J (2014). DSM-IV-TR Axes-I and II mental disorders in a representative and referred sample of unemployed youths: results from a psychiatric liaison service in a job centre. Eur Psychiatry.

[49] Sabatella F, von Wyl A Youth unemployment and mental health: An underestimated problem in Switzerland.

[50] Hammarström A, Janlert U (2002). Early unemployment can contribute to adult health problems: results from a longitudinal study of school leavers. J Epidemiol Community Health.

[51] Blum A (2015). Psychische Gesundheit Jugendlicher im betrieblichen Umfeld. Journal Gesundheitsförderung.

[52] Cohen J (1988). Statistical power analysis for the behavioral sciences, 2nd edition.

[53] Schulz P, Schlotz W, Becker P (2004). Trier Inventory for Chronic Stress.

[54] Fydrich T, Sommer G, Brähler E (2007). Fragebogen zur Sozialen Unterstützung: F-Soz-U; Manual.

[55] Dychko E (2016). 7 detailed steps for marketing your mobile app launch with content.

[56] Rice K (2013). How long does it take to build a mobile app?.

[57] Dimensional Research (2015). Failing to meet mobile app user expectations: A mobile app user survey.

[58] Kaul N (2015). First impressions count with mobile app quality.

[59] Reneflot A, Evensen M (2012). Unemployment and psychological distress among young adults in the Nordic countries: A review of the literature. Intl J Soc Welfare.

[60] McKee-Ryan F, Song Z, Wanberg CR, Kinicki AJ (2005). Psychological and physical well-being during unemployment: a meta-analytic study. J Appl Psychol.

[61] Paul KI, Moser K (2009). Unemployment impairs mental health: Meta-analyses. J Vocat Behav.

[62] Barak A, Grohol JM (2011). Current and future trends in Internet-supported mental health interventions. J Technol Human Serv.

[63] Fridrici M, Lohaus A, Glass C (2009). Effects of incentives in web-based prevention for adolescents: Results of an exploratory field study. Psychol Health.

[64] Viner R, Macfarlane A (2005). Health promotion. BMJ.

