# Supplementary material for: Can We Foster a Culture of Peer Support and Promote Mental Health in Adolescence Using a Web-Based App? A Control Group Study
Source: JMIR Ment Health. 2016 Sep 23;3(3):e45. doi: 10.2196/mental.5597 (PMC5074648; doi:10.2196/mental.5597)
Supplement: Multimedia Appendix 1 [file mental_v3i3e45_app1.pdf]

Appendix 1.

Table A. Mixed models for the prediction of change in chronic stress levels from pre- to postevaluation: Model A, B, and C (gender reference category—female, year—year of apprenticeship, year of apprenticeship reference category—first year).

**Predictor**	**Model A**			**Model B**			**Model C**		
	**Co-efficient**	**SE**	***P***	**Co-efficient**	**SE**	***P***	**Co-efficient**	**SE**	***P***
(Intercept)	1.39	0.04	<0.001	1.38	0.05	<0.001	1.38	0.05	<0.001
Group (Model B)	-	-	-	0.04	0.06	0.54	0.04	0.06	0.54
Group* Time (Model C)	-	-	-	-	-	-	-0.15	0.03	<0.001
Time	-0.15	0.03	<0.001	-0.15	0.03	<0.001	-0.04	0.07	0.56
Gender	-0.23	0.06	<0.001	-0.23	0.06	<0.001	-0.23	0.06	<0.001
Year	-0.14	0.06	0.01	-0.14	0.06	0.01	-0.14	0.06	0.01
Age	0.01	0.02	0.62	0.01	0.02	0.59	0.01	0.02	0.59

	**Random effects**			**Random effects**			**Random effects**		
		σ			σ			σ	
	Residual	0.20		Residual	0.21		Residual	0.21	
	Intercept	0.21		Intercept	0.21		Intercept	0.21	
