# Supplementary material for: Can We Foster a Culture of Peer Support and Promote Mental Health in Adolescence Using a Web-Based App? A Control Group Study
Source: JMIR Ment Health. 2016 Sep 23;3(3):e45. doi: 10.2196/mental.5597 (PMC5074648; doi:10.2196/mental.5597)
Supplement: Multimedia Appendix 2 [file mental_v3i3e45_app2.pdf]

Appendix 2.

Table B. Mixed models for the prediction of change in perception of social support from pre- to postevaluation: Model D, E, and F (gender reference category—female, year—year of apprenticeship, year of apprenticeship reference category—first year).

	**Model D**			**Model E**			**Model F**		
**Predictor**	**Co-efficient**	**SE**	***P***	**Co-efficient**	**SE**	***P***	**Co-efficient**	**SE**	***P***
(Intercept)	2.65	0.06	<0.001	2.68	0.06	<0.001	2.7	0.06	<0.001
Group (Model E)	-	-	-	-0.08	0.07	0.28	-0.15	0.09	0.08
Group* Time (Model F)	-	-	-	-	-	-	0.02	0.05	0.78
Time	0.06	0.04	0.148	0.06	0.04	0.15	0.15	0.09	0.12
Gender	0.13	0.07	0.08	0.12	0.07	0.09	0.12	0.07	0.1
Year	0.11	0.07	0.12	0.12	0.07	0.11	0.12	0.07	0.106
Age	-0.08	0.02	0.001	-0.08	0.02	0.001	-0.08	0.02	0.001
	**Random effects**			**Random effects**			**Random effects**		
		σ			σ			σ	
	Residual	0.4		Residual	0.4		Residual	0.4	
	Intercept	0.3		Intercept	0.3		Intercept	0.3	
